# A hermeneutic study of integrating psychotherapist competence in postnatal child health care: nurses’ perspectives

**DOI:** 10.1186/s12912-018-0311-1

**Published:** 2018-09-21

**Authors:** Katarina Kornaros, Sofia Zwedberg, Eva Nissen, Björn Salomonsson

**Affiliations:** 1 Department of Women’s and Children’s Health, Karolinska Institutet, Stockholm, Sweden; 2 Sophiahemmet Högskola, Stockholm, Sweden

**Keywords:** Aristotle, Child health services, Hermeneutics, Ideal type, Interprofessional relations, Nurses, Perinatal care, Psychotherapy

## Abstract

**Background:**

There is a considerable prevalence of and an increasing attention to emotional problems in families with infants. Yet, knowledge is scant of how to create efficient and accessible mental health services for this population. The study qualitatively explored public health nurses’ conceptions of a clinical project, in which psychotherapists provided short-term consultations and supervisions for nurses at Child Health Centres in Stockholm.

**Methods:**

In-depth interviews with fifteen nurses. The guideline of the interviews contained open-ended questions that were analysed applying a hermeneutical approach.

**Results:**

Four main themes crystallized; The nurses’ conceptions of their psychosocial work, Trespassing on another professional role, Interprofessional collaboration at the Child Health Centre, and The nurses’ conceptions of the psychotherapist’s function. In a second step, an analysis that clustered the nurses’ attitudes towards handling mental health problems yielded one last theme with three “Ideal types”; nurses who expressed “I don’t want to”, “I want to but I cannot”, and “I want to and I can” (take care of families’ emotional problems at the CHC).

**Conclusion:**

The nurses appreciated the easy referral and accessibility to the psychotherapists, and the possibilities of learning more about perinatal mental illness and parent-infant interactions. For a successful cooperation with the nurses, the therapist should be a team member, be transparent about his/her work, and give feedback about cases in treatment. The study also shows how the organization needs to clarify its guidelines and competence to improve psychological child health care. The paper suggests improvements for an integrated perinatal mental health care.

## Background

In Sweden, “Child Health Centres” (CHC) offer tax-funded primary health care to all children between 0 and 5 years. In contrast to, for example, the UK, Sweden uses separate units (“Maternity Clinics”, MVC) for prenatal health care. The MVC carries out pregnancy checks during pregnancy and prepares the parents for the birth. There is thus an overlap of the term “midwife”; in Sweden, she is only responsible up to the delivery including a one-time check-up a few weeks afterwards. The postnatal staff is called a CHC-nurse. The family chooses a local CHC and meets with the nurse, who tracks the children’s weight and length and examines their developmental progress.

In 1997, the National Board of Health and Welfare [1] provided guidelines for nurses to include CHC psychosocial support to families, based on the evidence that approximately 10–15% of new mothers show signs of postpartum depression (PPD) [2–4] and about 5–10% among fathers [5, 6]. Detecting parental distress can be difficult unless a family member signals it clearly to the CHC nurse, who may then refer to psychiatric care. This procedure, however, risks stigmatizing parents with babies who seldom regard themselves as ill in a psychiatric sense [7]. Health care routines in Stockholm demand that CHC nurses receive a brief training course in the Edinburgh Postnatal Depression Scale (EPDS) [8]. Screening is offered when the infant is 6–8 weeks, and the nurse talks with the mother about her questionnaire responses. This can lead to a few follow-up meetings and, if needed, a referral to an external general practitioner, psychologist, or a psychiatrist.

### Psychological treatment at the CHC

Although many studies indicate substantial results of parent-infant psychotherapy (PIP) [9–11] many cases of baby worries, a term comprising parental and infant emotional distress [12], risk being excluded from primary health care. The reason is the difficulty in detecting such problems and the lack of appropriate treatment at the CHC. The roadblock is threefold; a family in need will not voice their problems clearly, the nurse will not capture the signals sensitively enough, and she will not know how and where to refer the family for professional psychological help [13, 14].

To remedy this shortcoming, a method called “Short-term Psychodynamic Infant-Parent Interventions at Child Health Centres” (SPIPIC) was developed to integrate somatic and psychological health care [12]. It has two arms; a psychotherapist supervises CHC nurses and offers families short-term treatment on the premises. Supervisions aim to inspire nurses to detect and address baby worries. The therapies of 1–5 sessions aim to help parents tackle such issues and improve their contact with the baby. One author of this study introduced the method to a group of psychoanalysts trained in adult and parent-infant therapy. The Swedish Inheritance Fund provided funding and the clinical project took place 2013–2016.

This study is part of a research project evaluating the clinical project. The overarching aim is to investigate if SPIPIC might contribute to improving postnatal mental health care. The present paper focuses on the nurse’s experiences. When she observes baby worries, she is confronted with challenges; how to bring them up with parents and suggest adequate treatment. If a therapist becomes linked to the unit, the nurse needs to collaborate with him/her. Such integration of professional efforts might improve treatment quality, but factors that impede and facilitate this process need to be explored.

## Methods

### Aims

The study aimed to analyse CHC nurses’ previous experiences of taking care of families with baby worries. And, now that a professional psychotherapist was introduced, how did they experience being supervised by her, referring cases, and collaborating with her?

### Setting and sample selection

Six CHCs in Stockholm were selected to maximize the geographical and socio-economic variability. The centres hosted 3–8 nurses specialized in paediatric care, often also in public health. Each full-time nurse was responsible for about 350 children. The sampling procedure was as follows. Since we aimed to evaluate a clinical project, the interviewer (first author), approached all the involved CHCs. She asked the head nurse to supply names of colleagues who had a varying length of professional experience and professional positions. This resulted in fifteen nurses being interviewed, of which six were head nurses. All interviews were face-to-face. An unknown number declined to participate due to time constraints. The unknown number is due to the interviewer’s non awareness about how many nurses had been asked to participate. Yet, she got information about nurses rejecting the proposition. All participants were female, had Swedish background and their average age was 53 (35–65) years. Interviews took place during the end of the research project, January–May 2016. They were conducted by the first author who had profound experience in qualitative interviewing.

### Procedure

Interviews took place in each nurse’s consulting room. First, the interviewer went through the study aims and clarified routines of confidentiality. Characteristics about the interviewer was brought up before the interview started, such as reason for doing the research and personal goals. Participation was voluntary and if a nurse decided to leave the study, data would be deleted. All nurses consented by signing an agreement. Interviews lasted 1 hour and were audio-recorded and transcribed verbatim. The interview format was built on 14 questions concerning the nurses experience of perinatal mental health [PMH] and the clinical project. They were created by the first author and posed in an order that suited the topics broached by the nurse. When relevant, the interviewer could probe further into one area. Field notes were made at some occasions. Below are the interview questions:

1. Which were your expectations of the clinical project?
2. What kind of education do you have?
3. Have you felt any lack of any competence in your work?
4. How do you feel the collaboration with the psychotherapist worked?
5. Describe what happened in the supervision by the psychotherapist.
6. How did you experience the supervision?
7. How do you observe parents with emotional problems?
8. Tell me how you reason when you meet parents with babies who do not address that they need psychological help, yet you identify their problem.
9. Tell me if you experience that your ability to detect parents and children with emotional problems has changed during the project?
10. How do you experience bringing up questions about mental health with the parent(s)?
11. “What kind of” parents did you refer to the psychotherapist?
12. Did you mention to all parents you met that the therapist was working at the CHC?
13. How did you handle these cases before the psychotherapist was available here?
14. Does the project conform with your expectations?

### Data analysis

The analyses were based on philosophical hermeneutics [15], which presupposes that any interpretation of texts, words, or behaviours is created out of the respondent’s verbal and non-verbal testimonies as well as the researcher’s preconceptions. In the Geisteswissenschaften [16] or human sciences, of which this study is an example, the interpreter acknowledges his subjectivity and considers it essential for his/her interpretation. Interpretations will thus depend on the individualities of respondent and interviewer and will, accordingly, not reflect universal laws. Knowledge emerges in a dialogue based on equality between interviewer and informant in that both are human subjects [17]. True, analysing a text, in contrast to an interview, entails no dialogue but the interpreter maintains an internal discourse with the text and uses self-reflection to reach a deeper understanding. Each author of the paper, one social worker, two midwives, and one child psychoanalyst, applied this procedure to the interview texts and the group met regularly to discuss the material and the interpretations. We did not use any qualitative software since, in our opinion, it is more appropriate for example in content analysis. Our hermeneutic analyses were based on an interaction between discussions in the group and recourse to the interview transcripts.

The group analysed recurrent statements in the nurses’ comments and sought to detect their underlying meanings and collected them into themes that were labelled. This inductive analysis [18] was later complemented by an abductive approach [19, 20] of identifying Ideal types. Abduction involves forming conclusions from the information that is known. This procedure aimed at “making sense of data” ([20], p. 5) and at enabling further theorizing on the nurses’ attitudes in handling emotional family problems. Ideal types was introduced in sociology [21] and later in psychotherapy research [22, 23]. The researcher extracts concise descriptions out of each case and clusters observable phenomena and concepts in an interpretative or exploratory schema [22]. The clustering combines objective observations and the interviewer’s subjective emotional reactions to the interviewee [24].

## Results

All names contained in the result section are pseudonyms and have been changed to ensure the anonymity of study participants. Nurses from one CHC have identical initials, for example, Thea and Tora from CHC-T. The hermeneutic analysis crystallized four main themes. In a second step, an analysis that clustered the nurses’ attitudes towards handling mental health problems yielded one last theme.

### The nurses’ conceptions of their psychosocial work

Many nurses mentioned that health care organization had changed the last decades, with ensuing alterations in their duties. This created a tension between their previous medical training and today’s tasks, which increasingly concern psychological problems.

Dina: “I miss working with medical health care here (...). When the family doctor reform was instituted, all casualties disappeared. Earlier, we could get burns, wound dressing, suture removal and all that. It disappeared! Now they visit the family doctor instead.”

(The family doctor reform implied that all citizens should have the opportunity to choose a personal general practitioner). Nurses also described that this change meant that they must shift focus from the child to the parents. They reported that today’s parents need to be confirmed, their agenda is crammed, and they must achieve and compare themselves with others. Nurses felt they had become more of a “grandmother” or a “speaking partner”.

Paulina: “The number of families who feel mentally ill is incredibly larger than ten years ago…. A huge difference! One would think that today, every family is in a bad emotional state... Once you get a family without any worries, you almost think it’s a bit unusual.”

Some nurses pointed out that conversations during coffee breaks often revolved around distressed families and they deplored not finding time for exchange and reflection during working hours. Cleo missed time for reflection during supervisions:

“What will happen to me as well when I meet this mother who is so worried about her child (...) How do I feel then, how worried am I? How scared am I? What do I bring with me home? These are things I would like to leave behind at supervisions!”

Many nurses regarded the EPDS screening 6–8 weeks after childbirth as indispensable for capturing emotional issues. It helped them raise matters about mental illness in a less intrusive way. Most of them regarded the questionnaire score as a major source of understanding the mother’s emotional well-being. However, proceeding to suggesting to the mother some counselling sessions based on the EPDS responses, felt cumbersome.

Thea: “It gets to an entirely different level when you’re screening [EPDS]. Afterwards [when the results are discussed with the mother], you must have something to offer! We don’t have any therapeutic education or anything like that, but we have built on experience, quite simply, and if you don’t have that… then it must be hard!”

Some nurses abstained from EPDS-screening for mothers with insufficient knowledge of Swedish, or those who were problematic cases or difficult to identify, because they felt overloaded. They knew that these families needed psychotherapeutic help but did not refer them. They felt they had enough work to manage the usual tasks in time. The nurses also felt they needed more knowledge about parent-infant psychology and wanted introductory psychotherapy training. Some thought paediatric nursing education needed to be modernized.

Tanja: “Is [parental support] our job today? What does our education look like? There’s very little of [psychological issues] in our training. They may need to change [education] because it feels there will be more and more of this field. And then you would of course need to have someone like Therese [the therapist] (...) to guide [us]; is this [case] healthy or not healthy?”

Other nurses did raise psychological issues directly rather than waiting for the EPDS-screening or an explicit statement by the parent. Ebba “talked to herself” about how to interact with and address every family. Cleo used mother-infant interaction massage to discern attachment problems in the dyad.

Cleo: “In a group with parents (...) I can see someone with vast interaction difficulties, and I can ask [the mother]: ‘Is it difficult being close to the child this way?’ Maybe one mother always turns her child away from herself, and the child is turning outwards, away from her.”

In our interpretation, nurses who missed the medical tasks they had been trained in focused on the child’s physiological development and referred psychosocial problems instantly to the therapist or other professionals. They felt dismissed having been withdrawn from duties they felt capable of handling, such as ordinary medical problems. Instead, they felt forced to work with psychosocial issues, where they felt less competent. Others, who had changed focus or who had not previously worked according to the traditional medical role, deplored that they did not have the knowledge to work deeper with psychological problems.

### Trespassing on another professional role

Many thought that addressing the mother about her mental health was a sensitive topic. Hence, they separated the task of capturing her emotional problems from the therapist’s task of treating them. If this was not a possible option, many considered crossing this line to be an act of trespassing on another professional role.

Sara felt secure as a CHC-nurse but when encountering a sad mother in a EPDS-screening, she felt insecure and that she was “stepping into the therapist’s shoes”. Despite her training, she had not carried out EPDS-sessions for 15 years because she had felt like a “semi-psychologist” and “a little bluffer”. The reason for such feelings was not any lack of professional experience. Rather, some nurses felt they did not have anything new to say to the parent in the EPDS-sessions. When touching anxiety-provoking topics, they feared they might open up reactions they could not handle.

Erika (pretending to talk to a mother): “‘Okay, you're afraid to leave your home, because you're afraid someone will follow you, push your stroller out in the street and harm your baby’. I can’t explain WHY she has such thoughts! I can’t suggest what she can do. Then something else needs to be added.”

We interpret that Erika was stuck in a demand to offer the mother advice and suggestions. She was more familiar with having an active, advisory role and less trained in listening to and containing the parents affects, which was felt as trespassing on therapist territory. Having a psychotherapist at the CHC bridged this dilemma. She was now seen as a haven of wisdom and a relief for the nurse. Cases could be remitted to her and the therapist often received the cases without any reserve, which made them refer on a broader and less selective scale. Cases that were previously difficult to identify, or those which gave the nurse a “lump in the stomach”, could now be referred on a looser basis.

Cleo: “Some of my parents may not seem to be in a very bad mood or do not score very high on the EPDS. Yet, in our conversations I may find out that they are worried after all and are not feeling very well. And then I have told them that they have an opportunity to meet with a psychotherapist here”.

### Interprofessional collaboration at the child health Centres

Two CHC groups, positive and critical, crystallized. Ebba called the therapist “a godsend”, caring, structured, and involving the team in treatment decisions. Supervisions were regular, and the therapist provided transparent feedback about referred families. In our interpretation, Ebba addressed transparency in two ways; the therapist was open to the nurses’ points of view and, concretely, left her door open whenever possible. This also resulted in an open attitude between Ebba and the parents. She felt at ease asking the parents about therapies. At CHC-P, Pella described the therapist’s transparency:

“It is more enjoyable and easier to work with someone who completely trusts our confidentiality! She probably does, otherwise she wouldn’t tell us everything she’s actually doing! (...) We are all bound by professional secrecy. I think it's OK that she tells us everything when we ask for it. Such an approach inspires confidence, rather than someone saying, ‘No, I can’t tell you about it’.”

Sara had different experiences. The therapist Sonya was kind but “closed”. Sara received no information about referred families, since Sonya did not divulge anything. Supervisions took place monthly, but Sara felt they were “so-called supervisions”, since the therapist did not talk about the referred families. Sara called them “conversations about travelling and weather”. She requested clear rules of transparency and secrecy, trust in everyone’s competence, and focus on the families.

Sara: “I don’t know if Sonya wants to maintain confidentiality, maybe out of considerations of secrecy? I haven’t actually asked her, because when I asked about referred parents... [short pause] I didn’t get to know very much. I have respected that and thought she probably doesn’t want to say anything. Perhaps it’s right but it offers me less.”

One interpretation is that Sonya came from another professional context as a private practitioner and was used to sharing information more restrictedly, which clashed with the CHC culture. Nevertheless, the nurses appreciated her clinical work. The nurses were accommodating because they feared losing Sonya. Instead of bringing up the problems with her, they worked in parallel with her and found their own ways of complementing her administrative routines and secrecy.

On CHCs with less satisfactory supervisions and contacts with the therapist, parental discontent with therapy was interpreted as proof of the therapist’s failure. Still, no nurse raised such topics with the therapist. At CHCs where nurses were satisfied, discontent was brought up even though it was awkward. Pella informed the therapist about a dissatisfied parent:

“I must admit it was embarrassing, because in my eyes she’s good. You are working together, after all, she’s here once a week, we have supervision with her so she feels like a colleague. Such things are always tough to raise with a colleague”.

### The nurses’ conceptions of the psychotherapist’s function

Overall, nurses had vague ideas about how therapies were performed. At CHCs where supervisions were felt to be unsatisfactory, this added to the dissatisfaction and made nurses refer less to the therapist. In centres where supervisions worked well and the therapist was perceived as a team member, the “mystery” surrounding therapies was unimportant for their trust in her. In addition, these therapists gave some insight into how treatments were performed. In general, nurses were positive about the treatment model per se, even the ones who felt critical of the local psychotherapist.

Pella (positive of the local therapist): “It would be wonderful if this ended up being a project and became part of general child health care (...) especially in socio-economically burdened areas”.

Referrals to other units were expressed as time-consuming, since it implied an obligation to convince a professional that their patient was a case for them. In the case of GP consultations, some nurses felt the doctors did not have the necessary qualification. Thus, a local therapist made work more flexible and help more accessible. Families came for regular nurse calls followed by a therapy session. If the nurse was considering therapy for the family member(s), she could ask questions to the therapist, get immediate answers, and refer the family.

Tanja: “Sometimes, suggesting another unit to the family meets with a huge resistance. They seem to think it’s too much psychiatry and a harder step to go somewhere else. Here [to the CHC] they come anyway!”

Most parents were referred to the therapist because they brooded on their parental role and felt lonely and uncertain when interacting with the baby. Though many had a psychiatric history, such specialist units did not consider them as “their cases”. The nurses worried that their emotional states could affect the infant negatively and emphasized the importance of early intervention. Prior to the project, it had been difficult to implement this family approach at the CHC. In CHCs with many immigrants, nurses had difficulties in understanding the family member’s state due to language barriers, attitudes towards mental illness, and lack of familiarity with psychotherapy.

Pella: “It is a bit of our ‘mourning [problem] child’, or I think, it should be all of Sweden’s mourning child in some way. What do we do with [immigrant families] who do not crack all the [cultural] codes?”

Attitudes to taking care of psychological problems: Ideal Types.

The themes reported above resulted from analyses of the interviews; the nurses’ comments were grouped inductively into themes. Afterwards, we also analysed in another direction; nurses were clustered into Ideal Types according to their attitudes towards handling psychological problems. Three types emerged.

Ideal type 1: “I don’t want to”

Tora: “I am a CHC nurse. That's what I am! I have great tools for what I should be able to perform and for what my competence is supposed to include (…). There are limits to which dialogues we CHC-nurses can have with the parents. We are not therapists and we are not educated for it and then one should feel the limit of what a CHC visit implies”.

We interpret this nurse to be unwilling to expand her professional role to include working with mental health problems. She has decided not to work with what she feels amounts to a trespassing on another profession’s duties.

Ideal type 2 “I want to but I cannot”

Sandra: “This mom is so worried about every little thing. She had difficulties letting go of the child. In itself, that need not lead to a long-term psychological contact. It usually lights up at three-four months. I usually try to see if I can solve it myself as a nurse. But I felt nothing happened after our counselling meetings and phone calls… so I asked Sonya for an appointment. I couldn’t handle the mother’s worries.”

The nurse has understood that this mother is worried and has started consultations but feels she lacks skills to deepen the dialogue. She tries to understand the mother, but when she perceives that the mother’s worries do not diminish, her own worries about the mother increase. She questions her own capability of handling maternal worries.

Ideal type 3 “I want to and I can”

Ebba: “I have a supplementary assignment at CHC with groups of depressed mothers (...). I try with the help of infant massage and such to promote interaction and find the joy too! (...) Some [mothers] who are depressed can be very self-absorbed and need help opening up to see their little ‘Charlie’.

We interpret that the nurse has an interest and feels she can supervise such groups. She has noted that the mothers are self-absorbed and has found a solution to guide them to better understand their babies. She facilitates maternal self-insight by including the baby in the interaction.

## Discussion

This paper investigated nurses whose working place, Child Health Centres, had been provided with a psychotherapist through a clinical project. It integrated routine health care with qualified brief psychotherapy and aimed to enhance the competence and the incentive among nurses to handle perinatal psychological problems. It thus viewed the *nurse as a key person* in perinatal psychological health care. The discussion will cover [1] our study’s contribution to existing research on midwives’ and nurses’ attitudes to, and difficulties in dealing with, perinatal emotional issues, [2] the interview findings within an Aristotelian conceptual framework, and [3] conclusions from the interviews on how to organize and educate perinatal health care providers in ways that integrate medical and psychological perspectives.

### Comparison with other nursing studies

Some studies have focused on midwives’ and nurses’ attitudes to perinatal psychological problems. London midwives [25] indicated their willingness to take responsibility but they lacked confidence and knowledge. Many did not know about the “high incidence of perinatal mental health problems [and] the signs and symptoms of some of the most serious perinatal mental illnesses” (p. 334). Two studies of Australian midwives [13, 26] yielded similar findings. To improve the situation, Ross-Davie et al. [25] recommended better training and “adequate staffing levels and the provision of specialist perinatal mental health service for onward referral” (p. 334). McCauley et al. [13] suggested “improved liaison with other disciplines, [such as] a perinatal mental health nurse specialist or practitioner” (p. 792). Such a procedure was instituted in a Dublin project [27]. This differs from our project’s placement of a psychotherapist at the CHC, who used her expertise in treating parents and supervising nurses. We believe this is advantageous since depressed parents, as well as their nurses, are burdened by the fear of stigmatization when referred to other units. A therapist on the premises conveys, together with the CHC nurse, that *mental health issues are a natural and integral part of parenthood*. Also, a therapist’s expertise covers not only symptoms of emotional suffering but also how to create a fruitful dialogue with the patient. The need to deepen midwives’ skills in handling parental distress was shown by Rollans and co-workers [14]. They investigated qualitatively what took place at antenatal booking visits, in which midwives were required to make a routine psychosocial assessment. Their unfamiliarity was indicated by the many who read out the questions from their computer screen at the end of the visit, a procedure that might impact negatively on the future mother’s “comfort in disclosing her concerns and on her relationship with midwives and the maternity service” (p. 6). Midwives should thus be given “opportunities to discuss their own experiences of listening and responding to women’s trauma experiences” (p.7).

In our project, such opportunities were supposed to be given through the therapist supervisions. When such collaboration did not work well it was not at firsthand, in contrast to the London study [25], due to lack of knowledge of symptoms of perinatal distress and disorder. In fact, nurses in our project knew about such issues but were embarrassed when broaching them with the parents. This was reflected in several themes in the qualitative analysis, for example, that such dialogues with a parent felt like “trespassing” on the therapist’s profession. Her presence on site was much appreciated, but some nurses felt uncertain of how to utilize this opportunity. Judging from the interviews, a similar unfamiliarity probably existed among those therapists who seemed to have difficulties with maintaining professional secrecy while simultaneously being open with the nurses. This question will be further investigated in an upcoming interview study with the therapists.

Turning to our Ideal Types, we suggest that the one named “I want to but cannot” corresponds with many nurses in the studies from London and Australia. We found one reason behind such attitudes; the recent paradigm shift in health care, which deeply affected the nurses’ views of professional roles and pride; from medical tasks focusing on the child to a psychosocial family perspective. This made them feel uncertain about their professional duties. This finding might correspond with an argument in a British study [28] that nursing education has not realized that integrating knowledge in psychology, biology, and sociology is essential to promote holistic care. Possibly, when some nurses complained about insufficient time to counsel parents and reflect with colleagues, this might not only reflect hard facts but also uncertainties about their new professional role. When they were helped in this by the therapist they valued it greatly, whereas other nurses felt supervisions were unsatisfactory and unnecessary. Such differences did not relate to the therapists’ *clinical* skills but to her *openness* vis-à-vis the nurse team and her unclear ways of leading the supervision sessions. Too much attention was being paid to maintaining patient secrecy, and some therapists seemed unfamiliar with chairing the sessions. In contrast, their skills with patients were seldom questioned by the nurses. Everyone appreciated the clinical resource – although they knew little about the content of the therapies. Problems rather arose in the area of therapist-nurse *collaboration*.

### An Aristotelian framework

To further understand the nurses’ problems in handling baby worries, and to substantiate the validity of the Ideal types, we will discuss our results in line with Aristotelian epistemological concepts; *phronesis, techne,* and *episteme* [29]. *Phronesis* implies judgement based on practical wisdom and reflection used to approach the current situation. It has also been described as “gut feeling” or “tacit knowledge” [30, 31]. Skerrett [32] applies it to psychotherapeutic work and points out that to Aristotle, “our social practices constantly demand choices, such as how to be fair, how to take a risk, how to determine a course of action (...) Making the best choice demands wisdom” (p. 49). Phronesis also implies self-control, justice and courage. In Aristotle’s ([33], p. 95) own words, phronesis “is a true and reasoned state of capacity to act with regard to the things that are good or bad for man”. To illustrate, when a nurse sets out to consider a patient’s emotional state before inoculating, she applies phronesis.

*Techne* refers both to a technology adapted for a particular task and to a craft, for example, all the practical details involved in a vaccination; the procedure is the same regardless of the patient’s physical and emotional condition. *Episteme,* finally, refers to the logic behind an action. A nurse who decides, prior to a vaccination, to investigate if a child is infected applies episteme based on the general knowledge that it may be perilous to inoculate a sick child. The research that yielded such conclusions also took place within this paradigm.

To obtain a clinical knowledge that is as all-encompassing and profound as possible, it is essential to combine the three Aristotelian roads. Constrictions arise when one excludes or obfuscates any of them. In the Western health care paradigm, techne and episteme are viewed as essential for increasing knowledge and developing treatment methods. One example of the bias on episteme and techne was found in a Swedish study [34] comparing how nurses described their behaviour in parental groups with how they performed in video-recorded sessions. Despite their belief in the contrary, they tended to act as experts and leave little time to parents for discussions and reflection. The latter attitude seems much needed especially with parents with emotional problems. Similarly to the conclusions by Berlin et al., we believe that the therapists’ supervisions are essential to help the nurses develop in such a direction.

In contrast to techne and episteme, phronesis has not gained equal respect. It implies that we deliberate on the unique case, whereas episteme involves demonstration of the general. Therefore, only the latter paradigm has acquired the reputation of providing better treatment evidence. When taking care of patients, phronesis contributes by meeting the criteria of the individual. To substantiate, we bring in a phenomenologic study [35] where first-time mothers were interviewed about their experiences of support from the nurse. They appreciated moments when she showed patience with the mother, remained with her in difficult moments, and gave proactive support. Such behaviour seemed to reflect the nurse’s identification with the mothers, an attitude of “I know how it once was to be an inexperienced mother”, in other words one of phronesis. We argue that the panic and helplessness among the mothers in this study were similar to what an anxious or depressed mother may feel when being alone with her baby. We also claim that their nurses were able to use phronesis with them.

As a counterargument, one might claim that an attitude of phronesis will be attained as the nurse’s experience grows. Benner [36] describes the nurse’s gradual development to become an expert in her profession. A newly graduated nurse sticks to settled rules and routines, but with increased experience she can become more independent and trust her intuition. In Aristotelian terms, she has moved from a one-sided reliance on techne and episteme to including more of phronesis. Phronesis certainly needs time to develop, but the paradox in our study is that all nurses had a long professional experience. We suggest several factors that seemed to stunt their reflection, empathy and curiosity of the young families’ dilemmas; organizational factors, lack of training, and a change in today’s health care paradigm compared with the one prevailing during their training. As Kinsella and Pitman [37] point out, today’s medical education and politics prioritize “instrumental values”, a term close to techne. When the aims of phronesis, techne, and episteme conflict and this remains unacknowledged, problems will arise. Phronesis also implies to sometimes work in an atmosphere of uncertainty and even bewilderment, whereas in techne and episteme the answers seem more obvious. This conflict emerged in the attitudes among our nurses towards the EPDS-questionnaire. Like in other studies [30, 38], it was perceived as a user-friendly instrument. Nevertheless, many nurses feared handling the ensuing EPDS-sessions. Their apprehension of trespassing made them avoid talking about mental illness with the parents.

We wish to also interpret the ideal types in terms of the Aristotelian concepts. Type 1 nurses, “I don’t want to”, preferred to work with medical issues, instead of maintaining a holistic perspective of the family. They acknowledged psychological issues but delegated them directly to the “expert” psychotherapist. They did intuit emotional problems in the family but abstained from talking about them, seemingly because they should be addressed by professionals possessing relevant techne and episteme. Some acknowledged that personal life experiences had provided them with wisdom but claimed this was not possible to apply in conversations with the families. Thus, in not making full use of their phronesis, their “personhood”, “a crucial element in accomplishing the goals of medicine” ([39], p. 321) receded in the background.

Ideal type 2 nurses, “I want to but cannot”, believed treatment of mental illness requires an “unknown” mode of techne and episteme. They applied it to some extent but dared not to delve deeper. They devalued the very phronesis they felt they possessed. Ideal type 3, “I want to and I can”, seemed to find a balance in tasks demanding mostly techne and episteme (nutrition advice, weighing and measuring) and phronesis (handling EPDS-sessions and maintaining dialogues with the families).

Phronesis also implies *ethical considerations*. The nurses’ dilemma can be described in terms of “moral distress” [40, 41]. This concept covers situations when a professional knows the right thing to do, but the institution makes it difficult to do what s/he knows is best. Tiedje [41] highlights the risks when moral distress is not acted upon; it can overwhelm the nurse’s coping resources. Tiedje recommends a coach who listens, guides and sorts out the professional and emotional dilemmas. The therapists in our project seemed to assume this function in structuring supervisions and increasing the nurses’ psychological understanding. Moral distress can also be countered by courage [40], and the supervisors seemed to embolden nurses to bring up emotional problems with the parents.

### Perinatal health care organization and education

In this area, we emphasize the paradigmatic shift in nursing duties in recent years. Apart from differences between individual municipalities and county councils [42], we discovered “local CHC cultures” regarding attitudes towards psychological care. Paradoxically, this homogeneity at each CHC risks creating heterogeneity for families needing psychological help; they might not obtain adequate care if their needs fall outside the local culture.

Another finding is that the health care organization, though it demands nurses to focus on psychosocial issues, does not systematically train them in this. Perhaps, the organization does not clarify whether nurses should work according to the attitudes mirrored by the Ideal types 1, 2, or 3. When nurses declared they felt congested with duties and professionally disregarded, we interpret this to reflect their dissatisfaction with the gap between training and the organization’s requirements.

The psychotherapist’s patency and accessibility vis-à-vis the nurses seemed crucial for the group dynamics during supervisions. Nurses wanted her to lead yet also be a team member. Therapists were different, which led to divergent attitudes towards psychosocial issues among the CHCs. In general, the nurses’ views of the therapist depended on the head nurse’s attitudes towards her and the supervisions, but she rarely understood her importance in this.

Despite some nurses’ critique, they considered the therapist a needed resource. We suggest two interpretations; the nurses were eager to receive another therapist in the future and they also feared being openly critical of her. The nurses’ reluctance to criticize her was perhaps akin to their unwillingness to confront the health care organization with their discontent with the lack of time for reflection in peer group discussions and supervisions.

If a nurse seeks support for a mother from the Swedish psychiatric health care’s “first line psychiatry” [42], which is a low-level form of psychiatric care often instituted at GP clinics, this implies a detour in the referral process. The nurse interviews allow for an interpretation that integrating a psychotherapist at CHC implied faster referral and greater accessibility of specialised parent-infant treatment and, in addition, interchange of information between therapists and nurses.

As for immigrant families, they constituted a specific challenge in that the nurses felt a lack of cultural competence. This made them sometimes avoid bringing up such perceived problems with the family involved. This finding corresponds to previous research [30, 43]; the Swedish Child Health Care organization lacks competence and guidelines to deal with mental illness among immigrant families. Also, our therapists seemed unfamiliar with encountering patients from other cultures than their own. In today’s increasing immigration, we can expect mental illness to become more frequent among newcomers. Therefore, it is of importance to detect these problems and handle them at an early stage in a professional way.

### Limitations

The informants were sometimes recruited by the head nurse, which brings about the issue of voluntary participation. Possibly, some participants felt persuaded by her to be interviewed, but the interviewer emphasized that participation was voluntary and that one could withdraw at any time without the head nurse being informed. Perhaps, recruitment involved a bias in that the head nurse would only ask nurses whom she appreciated. If any such bias existed, it was hard to control for.

The hermeneutic analytic method, with its emphasis that the interpreter’s subjectivity forms part of the basis of the interpretation, raises the question of trustworthiness [44], including external validity. To maximize the level of trustworthiness, we created a group of four interpreters who worked, first individually and then together to reach reasonably coherent interpretations.

## Conclusions

Instituting early interventions for families with mental health problems is crucial for optimizing the child’s development. Psychotherapeutic interventions have proven to be effective in such situations. The challenge is how to detect these persons, talk with them about it, and offer high-quality care. We have evaluated one model where the psychotherapist was integrated with regular health care at the CHC. In general, nurses were positive and wanted it to be permanent due to the effective remittance procedure and easy accessibility, and the possibilities of learning more about perinatal mental illness and parent-infant interactions. This is urgent especially for nurses who either have little interest in PMH or are vigilant about such issues but feel poorly equipped to deal with them. For a successful cooperation with the nurses, the therapist should be a team member, be transparent about his/her work, and give feedback about cases in treatment. The study also shows how the organization needs to clarify its guidelines and competence to improve psychological child health care. It also needs to improve education in cultural competence regarding immigrant families. This applies both to nurses and therapists.

## Abbreviations

CHC: Child Health Centres
EPDS: Edinburgh Postnatal Depression Scale
PIP: Parent-infant psychotherapy
PMH: Perinatal Mental Health
PPD: Postpartum depression
SPIPIC: Short-term Psychodynamic Infant-Parent Interventions at Child Health Centres

## References

[1] Socialstyrelsen (1997). Stöd i föräldraskapet (Support in parenthood) SOSFS 1997:161.

[2] Gaynes BN, Gavin N, Meltzer-Brody S, Lohr KN, Swinson T, Gartlehner G (2005). Perinatal depression: Prevalence, screening accuracy, and screening outcomes Evidence Report/Technology Assessment AHRQ Publication.

[3] O'Hara MW, Swain AM (1996). Rates and risk of postpartum depression-a meta-analysis. Int Rev Psychiatry.

[4] Wickberg B, Hwang CP (1997). Screening for postnatal depression in a population-based Swedish sample. Acta Psychiatr Scand.

[5] Johansson M, Svensson I, Stenström U, Massoudi P (2016). Depressive symptoms and parental stress in mothers and fathers 25 month after birth. J Child Health Care.

[6] Paulson JF, Bazemore SD (2010). Prenatal and postpartum depression in fathers and its association with maternal depression: a meta-analysis. JAMA.

[7] Stern DN (1995). The motherhood constellation: a unified view of parent-infant psychotherapy.

[8] Cox J, Holden J, Sagovsky R (1987). Detection of postnatal depression: development of the 10-item Edinburgh postnatal depression scale. BJPsych..

[9] Cohen NJ, Lojkasek M, Muir E, Muir R, Parker CJ (2002). Six-month follow-up of two mother-infant psychotherapies: convergence of therapeutic outcomes. Infant Ment Health J.

[10] Murray L, Cooper PJ, Wilson A, Romaniuk H (2003). Controlled trial of the short- and long-term effect of psychological treatment of post-partum depression. 2. Impact on the mother--child relationship and child outcome. BJPsych.

[11] Salomonsson B, Sandell R (2011). A randomized controlled trial of mother-infant psychoanalytic treatment. 1. Outcomes on self-report questionnaires and external ratings. Infant Ment Health J.

[12] Salomonsson B (2018). Psychodynamic interventions in pregnancy and infancy: clinical and theoretical perspectives.

[13] McCauley K, Elsom S, Muir-Cochrane E, Lyneham J (2011). Midwives and assessment of perinatal mental health. J Psychiatr Ment Health Nurs.

[14] Rollans M, Schmied V, Kemp L, Meade T (2013). ‘We just ask some questions...’ the process of antenatal psychosocial assessment by midwives. Midwifery.

[15] Gadamer HG (1975). Truth and method.

[16] Dilthey W. Introduction to the human sciences. Makkreel R, Rodi F, editors. Princeton, NJ: Princeton University Press; 1989.

[17] Gill S (2015). “Holding oneself open in a conversation” – Gadamer’s philosophical hermeneutics and the ethics of dialogue. J Dialogue Stud.

[18] Thomas DR (2006). A general inductive approach for analyzing qualitative evaluation data. Am J Eval.

[19] Kloesel C, Houser N (1998). The essential Peirce, vol. 2: 1893–1913.

[20] Tavory I, Timmermans S (2014). Abductive analysis: theorizing qualitative analysis.

[21] Weber M (1904). Die “Objektivität” sozialwissenschaftlicher sozialpolitischer Erkentnisse (“objectivity” in social science and social politics). Gesammelte Aufsätze zur Wissenschaftslehre.

[22] Kächele H, Schachter J, Thomä H (2009). From psychoanalytic narrative to empirical single case research.

[23] Wachholz S, Stuhr U (1999). The concept of ideal types in psychoanalytic follow-up research. Psychother Res.

[24] Lindner R, Fiedler G, Altenhofer A, Götze P, Happach C (2006). Psychodynamic ideal types of elderly suicidal persons based on counter transference. J Soc Work Pract.

[25] Ross-Davie M, Elliott S, Sarkar A, Green L (2006). A public health role in perinatal mental health: are midwives ready?. Br J Midwifery.

[26] McCann T, Clark E (2010). Australian bachelor of midwifery students’ mental health literacy: an exploratory study. Nurs Health Sci.

[27] Madden D, Sliney A, O’friel A, McMackin B, O’callaghan B, Casey K (2018). Using action research to develop midwives’ skills to support women with perinatal mental health needs. J Clin Nurs.

[28] Mowforth G, Harrison J, Morris M (2005). An investigation into adult nursing students’ experience of the relevance and application of behavioural sciences (biology, psychology and sociology) across two different curricula. Nurse Educ Today.

[29] Garefalakis J (2004). Paideia: Om bildningens historiska rötter (paideia: on the historical roots of “Bildung”).

[30] Borglin G, Hentzel J, Bohman DM (2015). Public health care nurses ‘views of mothers’ mental health in paediatric healthcare services: a qualitative study. Prim Health Care Res Dev.

[31] Belle MJ, Willis K (2013). Professional practice in contested territory: child health nurses and maternal sadness. Contemp Nurse.

[32] Skerrett K (2016). We-ness and the cultivation of wisdom in couple therapy. Fam Process.

[33] Aristotle. *Nicomachean ethics*. Kitchener, Ont.: Batoche Books; 1999.

[34] Berlin A, Rosander M, Frykedal KF, Barimani M (2018). Walk the talk: leader behavior in parental education groups. Nurs Health Sci..

[35] Hong TM, Callister LC, Schwartz R (2003). First-time mothers’ views of breastfeeding support from nurses. MCN Am J Matern Child Nurs.

[36] Benner, P (1984) From novice to expert: Excellence and power in clinical nursing practice. AJN: Dec 1984, Vol 84, Issue 12, pp 1480.

[37] Kinsella EA, Pitman A (2012). Engaging phronesis in professional practice and education. Phronesis as professional knowledge.

[38] Rush P (2012). The experience of maternal and child health nurses responding to women with postpartum depression. Matern Child Health J.

[39] Boudreau J, Fuks A (2015). The humanities in medical education: ways of knowing, doing and being. J Med Humanit.

[40] Gallagher A (2011). Moral distress and moral courage in everyday nursing practice. Online J Issues Nurs.

[41] Tiedje LB (2000). Moral distress in perinatal nursing. J Perinat Neonatal Nurs.

[42] Kartläggningsrapport. Uppdrag Psykisk Hälsa. Kartläggningsrapport: Första linje för barn och ungas psykiska hälsa. En kvantitativ beskrivning utifrån data insamlad mars-september 2014. (Report: First line mental health of children and adolescents. A quantitative description based on data collected March-September 2014). Retrieved 180520. Available from: https://www.uppdragpsykiskhalsa.se/fl/.

[43] Berlin A (2010). Cultural competence in primary child health care services - interaction between primary child health care nurses, parents of foreign origin and their children.

[44] Lincoln YS, Guba EG (1985). Establishing trustworthiness. Naturalistic inquiry.

